# Pilot Study: Magnetic Motion Analysis for Swallowing Detection Using MEMS Cantilever Actuators

**DOI:** 10.3390/s23073594

**Published:** 2023-03-30

**Authors:** Johannes Hoffmann, Sebastian Roldan-Vasco, Karolin Krüger, Florian Niekiel, Clint Hansen, Walter Maetzler, Juan Rafael Orozco-Arroyave, Gerhard Schmidt

**Affiliations:** 1 Department of Electrical and Information Engineering, Faculty of Engineering, Kiel University, 24118 Kiel, Germany; 2 GITA Lab, Faculty of Engineering, Universidad de Antioquia, Medellín 050010, Colombia; 3 Faculty of Engineering, Instituto Tecnológico Metropolitano, Medellín 050536, Colombia; 4 Fraunhofer Institute for Silicon Technology ISIT, 25524 Itzehoe, Germany; 5 Department of Neurology, Kiel University, 24118 Kiel, Germany; 6 Pattern Recognition Lab, Friedrich-Alexander-Universität, 91054 Erlangen, Germany

**Keywords:** cantilever actuator, digital signal processing, magnetic motion sensing, MEMS, swallowing, swallow detection

## Abstract

The swallowing process involves complex muscle coordination mechanisms. When alterations in such mechanisms are produced by neurological conditions or diseases, a swallowing disorder known as dysphagia occurs. The instrumental evaluation of dysphagia is currently performed by invasive and experience-dependent techniques. Otherwise, non-invasive magnetic methods have proven to be suitable for various biomedical applications and might also be applicable for an objective swallowing assessment. In this pilot study, we performed a novel approach for deglutition evaluation based on active magnetic motion sensing with permanent magnet cantilever actuators. During the intake of liquids with different consistency, we recorded magnetic signals of relative movements between a stationary sensor and a body-worn actuator on the cricoid cartilage. Our results indicate the detection capability of swallowing-related movements in terms of a characteristic pattern. Consequently, the proposed technique offers the potential for dysphagia screening and biofeedback-based therapies.

## 1. Introduction

The normal swallowing process involves the activation of more than 30 pairs of muscles to intake food, liquids, and saliva: a process mediated by central pattern generators within the brain stem. This process is characterized by a well-defined sequence of phases, i.e., pre-oral and oral, pharyngeal, and esophageal. Functional or structural alterations can produce swallowing dysfunction, also known as dysphagia, which can produce, for example, malnutrition, dehydration, and aspiration of the meal to the airway [1,2]. Dysphagia is a syndrome produced by a wide spectrum of diseases and conditions with especially high incidence and prevalence in elderly people suffering from neurological diseases, such as Parkinson´s disease, multiple sclerosis, muscular dystrophy, and dementia [2,3].

The instrumental diagnosis of dysphagia is performed by two techniques: videofluoroscopy and fiberendoscopy. These reference methods for dysphagia diagnosis are associated with unwanted side effects and complications [4]. For example, videofluoroscopy implies X-ray exposure with collateral risk of induced cancers [5], and fiberendoscopy is an invasive assessment where the endoscope is introduced to the larynx, which is uncomfortable and prone to gagging, vomiting, and more rarely, complications such as laryngospasms [6]. Furthermore, both techniques lack standardized protocols and scoring systems, which make them relatively subjective and expert-dependent [7]. These limitations have motivated the search for non-invasive and objective strategies to diagnose and screen swallowing disorders [8], such as biosignal-based approaches.

Accelerometers have been used experimentally to establish differences between normal and abnormal swallows from features extracted considering different domains (time, frequency, time-frequency, and nonlinear dynamics) [9,10]. These analyses have been performed using statistical comparisons, machine learning, and deep learning models [9,11,12]. Such studies suggest that the vibration analysis can help to estimate the duration of different swallowing-related events accurately, e.g., the opening and closure of the upper esophageal sphincter [13]. The CA is intended to describe swallowing from a mechanical point of view, but it has been routinely acquired in combination with microphone sounds. The latter allows the analysis of swallowing in an acoustic way, and information collected from both sources are complementary and not interchangeable [14]. Such an acoustical *dimension* of swallowing has also been explored with digital stethoscopes, but the low-pass effect of the diaphragm limits the spectral analysis to frequencies below 1 kHz [15]. On the other hand, speech–language pathologists widely use the perceptual evaluation of voice quality during clinical swallow examinations because it gives valuable clues regarding swallowing malfunctioning [16,17]. Even though speech production and swallowing are strongly linked at anatomical and physiological levels [18,19,20], few studies have addressed this association. Although significant statistical differences have been found between features extracted from speech in healthy individuals and dysphagic patients [21], the results have not yet been standardized. Additionally, the association between the voice changes perceived by the pathologists and the variations observed in different computed features remains unclear.

Several studies have explored the use of surface electromyography for dysphagia evaluation and biofeedback, based on electrophysiological information [22,23,24]. Nevertheless, the majority of these studies are mostly descriptive and focused on differences in amplitude and duration of normal/abnormal swallows, with some exceptions related to the use of automatic algorithms for detection of onset and specific swallowing phases [25,26,27,28,29,30]. Other sensors have been tested to detect swallows, such as bioimpedance, electromyography [25,28], nasal airflow [31,32], mechanomyography [27,32], and piezoelectric sensors, which all have shown the capability of swallow detection and dysphagia evaluation [33,34].

Nevertheless, none of the above sensors and biosignals have been successfully implemented in clinical routine, mainly due to the lack of standardization, validation, and limitations related to the evaluation of clinical aspects that reference methods allow to assess. As a result, the evaluation of new types of sensors for swallowing evaluation and biofeedback is still an open research field.

Magnetic sensors are applied for motion sensing in inertial measurement units (IMUs) by detecting a rotation in the geomagnetic field [35,36]. This method can be transferred to arbitrary sources such as permanent magnets, which enable application-specific motion-sensing solutions (such as tongue tracking [37]). However, full reconstruction of position and orientation in the 3D space requires a multitude of sensors and actuators to avoid ambiguity [38,39,40]. Derived metrics, such as fundamental frequencies or relative changes in position (for a temporarily fixed orientation), might still be obtained in a more limited setup [41]. Such setups with multiple actuators require a separation of signals during motion with multiplexing schemes (i.e., by frequency, time, or spread code). Corresponding sensors might be selected based on the available bandwidth and frequency-dependent magnetic noise density. Potential candidates for motion-related biomedical applications include various types of well-established magnetometers such as fluxgate sensors [42], magnetoresistive (MR) sensors [43], and magnetoimpedance (MI) sensors [44], as well as more experimental magnetoelectric (ME) sensors [45]. While coils can be used as magnetic actuators with arbitrary signals, they suffer from high power consumption due to copper resistance, which is detrimental for most wearable applications. Oscillating cantilever actuators with permanent magnets offer a potential alternative for the generation of AC fields [46]. Recent research indicates significant energy/range benefits of cantilever actuators in multiple fields, such as communication [47] or magnetic excitation [48]. Consequently, microelectromechanical systems (MEMS) cantilever actuators seem especially promising for biomedical applications due to the small form factor (wearable, array-capable). Therefore, the goal of this work is to analyze the suitability of MEMS for non-invasive and quantitative swallowing evaluation in terms of a novel magnetic approach for dysphagia screening.

## 2. Materials and Methods

### 2.1. Cantilever Actuators

The proposed setup uses MEMS, which were originally designed as magnetic field sensors. Each device consists of a cantilever (one-side clamp to a substrate, length: 1.5 to 2 mm) with a 4×4 array of permanent magnets (wafer level integrated). More information on these, including a detailed schematic, can be found in [46]. In the sensor role, magnetic field gradients cause a proportional force (torque in consequence) which results in an oscillation of the cantilever. A piezoelectric layer transforms the mechanical stress into charge, which is then amplified and read out. The mechanical system corresponds to a resonator with a frequency range of 1 to 2 kHz and a quality factor of a few hundred for the devices selected for the subsequently described experiments (see Section 2.2 for details).

**Figure 1:**
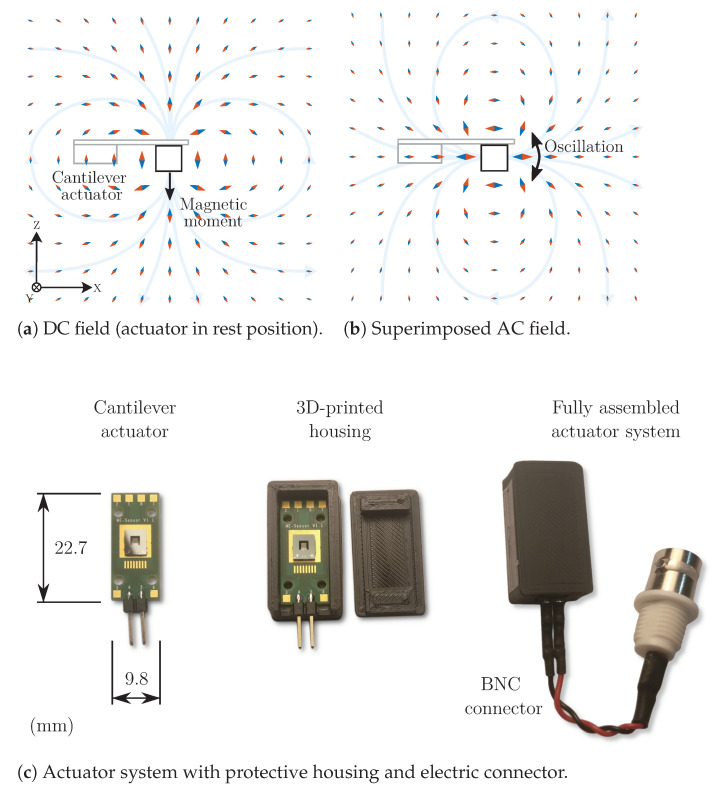
Overview of the principle and the physical setup of the cantilever actuator system.

The inverse operation scheme (actuation instead of sensing) employs the integrated permanent magnets as a source of a magnetic field. In the stationary case, a dipole vector field is assumed with the magnetic moment oriented in the negative z direction (Figure 1a).

The red arrow tips indicate the direction and absolute value (logarithmic size) of the corresponding magnetic flux density in the x-z plane. An inverse magnetic moment (e.g., reversed magnet) yields the same absolute field value with inverse direction (blue tips). The dipole approximation is also commonly used for other field sources, such as cylindric coils, which can be fed by a sinusoidal current to generate an equivalently shaped field where the amplitude switches periodically between the indicated directions.

In a similar way, the cantilever actuator might be driven by feeding an alternating voltage to the electrodes. Consequently, mechanical stress caused by the piezoelectric layer results in an oscillation of the cantilever (and the attached magnets). This oscillation is assumed to be mainly a tilt around the y-axis by low single-digit degrees. For a sinusoidal rotational movement with an exemplary peak value of ±1${}^{{}^{\circ}}$, the DC magnetic field from the stationary case is superimposed by a weak magnetic AC field (Figure 1b), whose dipole-like shape is oriented perpendicular to the DC field and the axis of rotation. This AC component was computed for visualization by subtracting the DC field (0${}^{{}^{\circ}}$ tilt) from the values obtained for the extremal points (±1${}^{{}^{\circ}}$ tilt). The resulting values and directions for both positive and negative half-wave are again indicated by red and blue arrow tips. For a maximum ±1${}^{{}^{\circ}}$ tilt, the AC field exhibits an amplitude like a dipole with 1.8% magnitude of the DC field.

For practical purposes, excitation at the resonance frequency was chosen to maximize the flux density of the alternating field. As a uniaxial magnetic sensor is used, the orientation of the sensor towards the actuator also constrains the achievable magnetic flux density in the experiments.

The complete actuator system (Figure 1c) consists of a printed circuit board (PCB) with the cantilever element, which is applied in a protective box made out of 3D-printed material (ABS) with a connector for the excitation signal.

### 2.2. Measurement Setup

**Figure 2:**
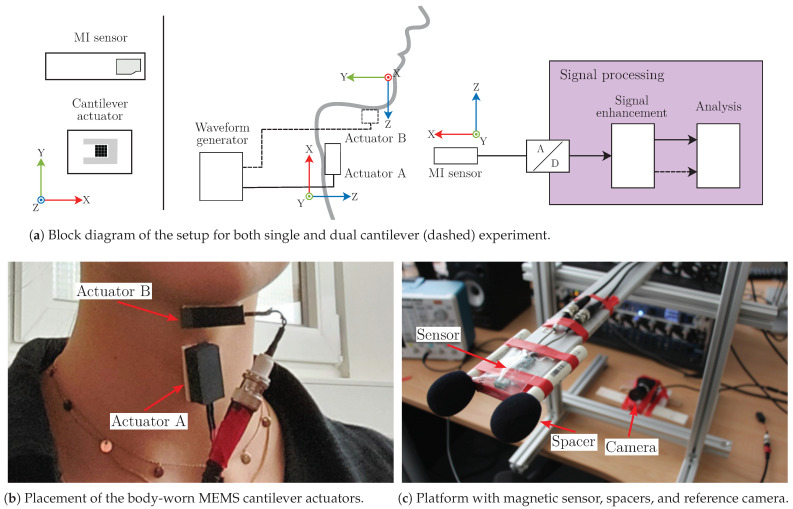
Overview of the experimental setup for magnetic swallowing assessment.

For our magnetic motion analysis, the movement during the swallowing process was captured by attaching one or more magnetic sources on the throat and detecting the emitted signal using magnetic field sensors. The measurement setup comprised up to two magnetic sources and a uniaxial magnetic field sensor as depicted in Figure 2a. The actuators were powered by an arbitrary waveform generator (Tektronix AFG 31022) with sine signals of different peak-to-peak voltages $V_{a/b}$ at their respective resonance frequencies $f_{\mathrm{res},a/b}$. Actuator A ($f_{\mathrm{res},a}$ = 1397 Hz, $V_{a}=$ 20 Vpp) was used for all single channel experiments. Actuator B ($f_{\mathrm{res},b}$ = 1039 Hz, $V_{b}=$ 2 Vpp) was only employed as a secondary source for the subsequent dual actuator experiment and is therefore displayed with a dashed line in Figure 2a.

A commercial magnetoimpedance (MI) sensor (Aichi Steel MI-CB-1DJ) was used, which claims detection capability of nT-range magnetic fields with an equivalent magnetic noise density below 100 pT/$\sqrt{\mathrm{Hz}}$ between 0.1 and 10 kHz, a sensitivity of 5 V/μT, and a 3 dB-bandwidth of 10 kHz. Analog to digital conversion of sensor signals was performed at a sample rate of 25.6 kHz by a 24 bit voltage input card (NI 9239 + NI cDAQ-9174 chassis) with an input range of ±10 V. The device was connected to a measurement PC, where further processing steps were applied in real time (see Section 2.4). The sensor itself was supplied by a single-channel DC voltage source (Korad KWR103) at +15 V. All components were connected by coaxial BNC cables.

### 2.3. Data Acquisition

**Figure 3:**
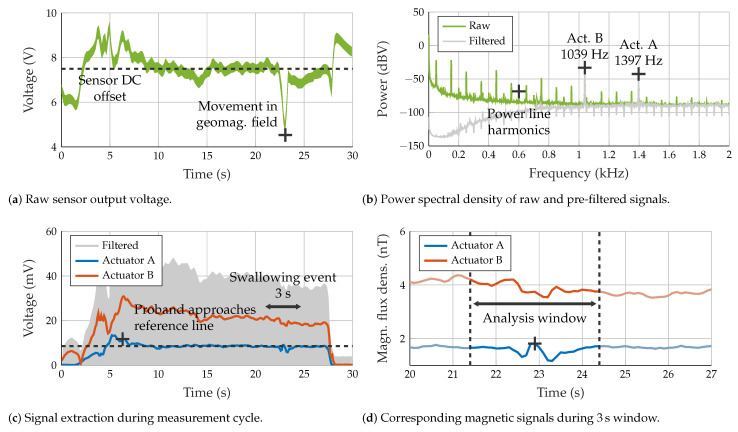
Functional overview of the signal enhancement pipeline.

The MI sensor was secured on an adjustable aluminum rack adapted to the subject’s height (Figure 2c). Besides the sensor, two spacers were adapted to maintain the neck in an upright position, aiming to capture the swallowing movements as naturally as possible and to reduce motion artifacts. A synchronized camera was used to record the movement of the throat as a reference for swallowing detection. One actuator was placed in a protective box attached to the subject’s neck at the level of the cricoid cartilage (Actuator A in Figure 2b). This point is recommended to acquire sound and vibration-related signals [49,50], especially for analysis of the pharyngeal phase (the larynx elevation pulls this cartilage up and forward [51]). The subjects observed real-time feedback of the signal in terms of an on-screen reference line (dashed line in Figure 3c) in order to maintain the basal signal level close to such a reference line. This feedback helped to maintain a similar position and distance between the actuator and the MI sensor to obtain reproducible and comparable measurements.

Table 1 shows specifications of three healthy subjects who performed this experiment. The measurement protocol consisted of three different swallowing tasks: dry swallows (saliva), 20 mL of water, and 20 mL of mild yogurt (fat: 1.5%, density: 1.027 g/mL). Each subject conducted each task 3 times (9 trials per subject, 27 trials overall). These swallowing tasks are routinely performed in the clinical bedside swallow examinations [52], and they have differential muscle activation patterns in terms of duration and amplitude [53]. The volumes were selected according to the “dysphagia limit” in healthy individuals found by Aydogdu et al. [24], i.e., the value above which piecemeal deglutition appears (20 mL).

We also performed an extension of this experiment to determine the regularity of the measures provided by actuators placed in different positions of the neck. We placed an additional actuator centered underneath the lower jaw on the suprahyoid muscles (Actuator B in Figure 2b). In this additional experiment, only the intake of 20 mL of water (6 trials) was assessed in one healthy subject (female, 24 years old). The muscles of the evaluated region participate in the tongue and jaw stabilization during the oral phase [51] and in the pharyngeal one for larynx and hyoid bone elevation to protect the airway [54]. The Actuator A was again used to align the instantaneous signal and the reference line.

### 2.4. Pre-Processing

Generally, the aggregated sensor output signal for our scenario contained up to two (dual actuator setup) desired signal components as well as undesired signals (e.g., power hum or thermal noise). For further analysis steps, the desired components were extracted. The applied processing chain was based on Hoffmann et al. [41] with a different parametrization due to the chosen sensors and actuator types. As the display of the reference line during the experiment required a feedback mechanism, a slightly simplified real-time implementation of the following processing chain was used. Results for presentation in this paper were processed offline with zero-phase filters.

Figure 3 shows the fundamental processing steps which start with the raw digitized sensor voltage signal from the data acquisition card. This signal was located around the sensor DC offset (single-end voltage supply) and was also affected by magnetic signals induced by motion in the geomagnetic field (Figure 3a). A Butterworth highpass filter (2nd order, 800 Hz) was applied to reject low-frequency noise. Afterward, undesired power net hum (fundamental frequency and harmonics) was suppressed by an IIR comb filter (512th order, 3 Hz bandwidth), which corresponds to a notch spacing of 50 Hz for a sample rate of 25.6 kHz. Spectral input and output signals for both processing steps are displayed in Figure 3b.

The resulting voltage signal was then converted to the equivalent magnetic signal. As the employed MI sensor features an almost constant frequency-dependent sensitivity curve in the region of interest, a division by the conversion factor of 5 V/μT was sufficient, and no further equalizing was required.

Depending on the number of actuators used, the signal was forwarded to one or two separate paths where 2nd order Butterworth bandpass filters were applied with cutoff frequencies centered at ±200 Hz around the desired excitation frequency.

The final noise reduction step was performed by a matched filter in a correlator realization (complex demodulation with subsequent integration). The integration time was empirically set to 100 ms which yields a bandwidth of 10 Hz for the resulting signal. The phase was dropped, as only the magnitude was of interest here. Figure 3c visualizes this fundamental acquisition process where the subject tries to converge the demodulated signal (Actuator A) towards the reference line. The subject induced swallowing once the match between them was produced.

### 2.5. Segmentation and Detrending

After data preprocessing, the swallowing events in the signal were segmented by selecting a window of ±1.5 s centered in the local maximum of the signal while swallowing (Figure 3d). For all 27 swallowing events with 1 actuator, an offset removal was conducted by using highpass filtering according to [55] (filter order of twice the sample rate). The separation of the swallowing events for the dual actuator experiment was conducted by choosing the maximum of the same signal produced by Actuator A. The trend removal was performed separately for both signals in the same manner as for the single mode.

### 2.6. Signal Characterization

Features in time, frequency, and time-frequency domains were extracted from each signal. The root mean square (RMS) and variance (VAR) were computed in the time domain. The mean frequency (MNF), median frequency (MDF), mean power (MNP), and peak frequency (PKF) were obtained in the frequency domain. Table 2 shows the mathematical formulations according to Phinyomark et al. [56]. Additionally, the frequencies where the spectrum drops 3 dB below the reference line were computed, denoted as $f_{\mathrm{low}}$ and $f_{\mathrm{high}}$. The difference between them, i.e., the power bandwidth (BW) was also extracted.

Moreover, we performed a wavelet decomposition in the time–frequency domain. Wavelet-based features have been extracted from accelerometry, sound, and surface electromyography signals for the detection of swallowing-related events, characterization of healthy and non-healthy swallows, and detection of swallowing phases, among others [9,57,58,59]. In this way, we computed the energy of the approximation and decomposition coefficients. We selected the db3 mother wavelet ad hoc, with four decomposition levels. Table 2 shows mathematical formulae.

Correlation matrices were used to quantify similarities between individual swallowing signals. Therefore, the power-normalized cross correlation for all available signal pairs was computed. The maximum value of each resulting correlation signal was displayed at its corresponding position in a 2D matrix plot. The results for a total of 27 swallowing events for the single actuator experiment (3 subjects, 3 tasks, 3 trials) were grouped per swallowing task and subject. In an equivalent approach, a second correlation matrix for Subject “b” was computed to compare the nine swallowing events for this subject (from the first experiment) with the additional six (from the dual actuator experiment).

## 3. Experiments and Results

### 3.1. Visual Inspection

**Figure 4:**
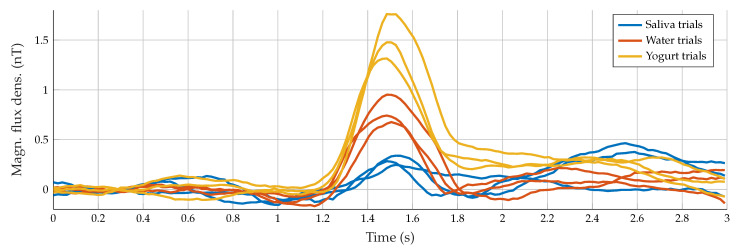
Example signals of all swallowing trials with different liquids for Subject “b”.

**Figure 5:**
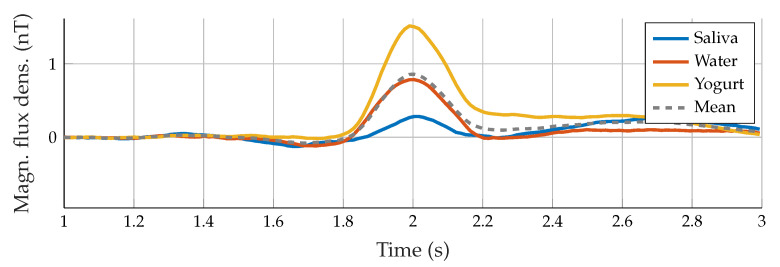
Example of the average signals for each swallowing task of Subject “b”.

For all results of the three participants, the swallowing process was clearly detectable in the measured motion signals. Furthermore, repetitive visual patterns were observable, which can be seen in Figure 4 and Figure 5, where all trials with one actuator for Subject “b” are depicted. In theory, each subject reached the reference signal before starting the swallowing movement and did not perform more movements than desired.

**Figure 6:**
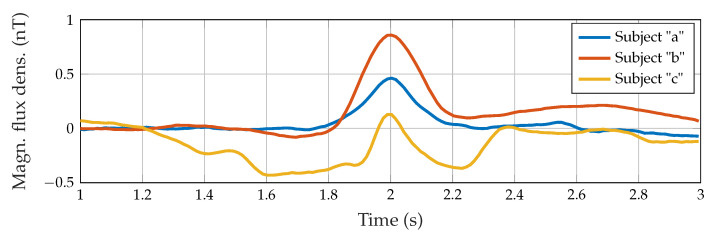
Prototype swallowing signal for each subject.

In practice, movement-related artifacts were produced, and matching the signal and the reference line was quite difficult. Therefore, all signals were demeaned using the first 50 ms as a reference for better visual comparison and subsequent alignment between all measurements. A maximum amplitude between 0.25 and 1.8 nT indicated swallowing activity, which matched the time when the movement of the throat was observable in the video. The duration of the entire swallowing process differed between 80 and 200 ms and was partially masked by movement artifacts. To characterize the measured signals and to distinguish between the swallowing process of different subjects, the peak-to-peak amplitude, as well as the pulse width, were calculated. This feature was defined by choosing half of the amplitude as a reference for the duration calculation. Each subject showed subtle individual characteristics in the signal and visual similarities for all three swallowing tasks (saliva, water, and yogurt). Figure 6 shows subject-specific prototype signals created by averaging the three tasks performed per participant. Note that each subject retrieved prototype signals with different properties, i.e., there was a subject-dependent behavior.

**Figure 7:**
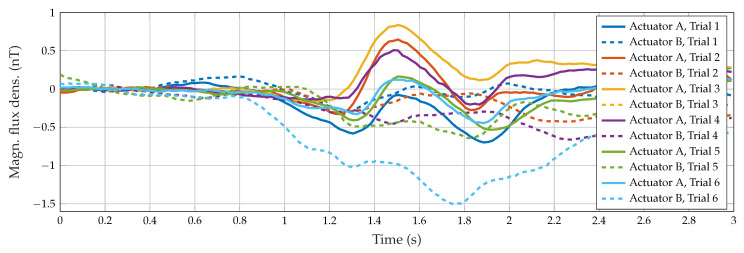
Signals of both actuators for six swallowing attempts with water.

From the second experiment with the dual actuator setup (Subject “b”, six additional trials of water intake), we evaluated the consistency between data provided by both actuators (Figure 7). The swallowing pattern can be further validated: for all nine swallowing trials for Actuator A, a pulse width of 33.68 ms with a standard deviation of 1.00 ms occurred. The mean amplitude was 0.79 ± 0.28 nT. For the assessed subject, the amplitude exhibited a clear difference between swallowing tasks: 0.35 ± 0.07 nT for saliva and 1.49 ± 0.23 nT for yogurt. These results are also visible in Figure 5. Otherwise, consistent measures were found in data from Actuator B, despite the sixth trial which behaves as an outlier.

### 3.2. Signal Characterization

The swallowing-related signals exhibited the highest power in low spectral components (below 10 Hz). The average mean frequency was 0.86 ± 0.23 Hz, and the median frequency was 0.48 ± 0.31 Hz. Moreover, the signals were very smooth, so the classical spectral analysis is not helpful to characterize the collected signals.

**Figure 8:**
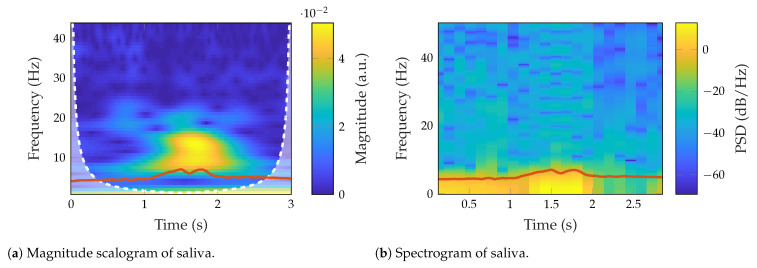
Spectral examples of the saliva-related signal in the single actuator experiment.

Figure 8 shows an example of the magnitude scalogram and the spectrogram of a dry swallow in the single actuator experiment for Subject “a”. The actuator output is highlighted in red for both scalogram (Figure 8a) and spectrogram (Figure 8b). The color bar shows the power spectral density (PSD). Despite the low-frequency vibration produced during swallowing, the power increase during the precise moment of swallowing is noticeable. Furthermore, there is a slight increase in the frequency components. This result was observed in all trials and swallowing tasks, as well as for the single and dual actuator experiments. Thus, our setup was capable of detecting variations produced by swallowing under different consistencies.

We performed an analysis to evaluate the inter-task variability; Table 3 provides features that may be suitable to detect differences between boluses. Moreover, Table 4 illustrates the variations between cantilevers in the additional experiment with the dual actuator setup.

### 3.3. Correlation Matrix

**Figure 9:**
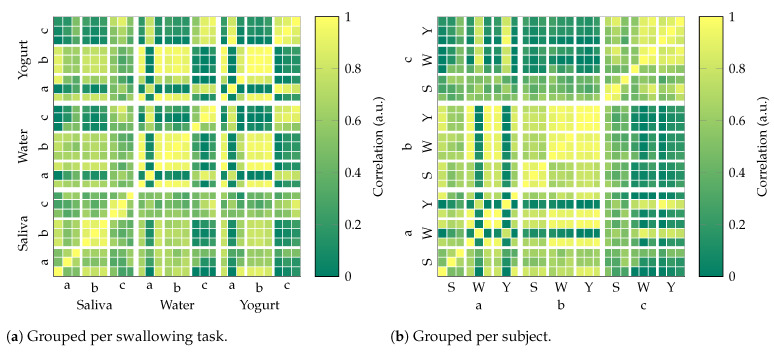
Normalized cross-correlation for each of the 27 trials grouped by different categories.

Figure 9 compares all nine trials (three per subject) for each swallowing task (saliva, water, yogurt) of the single actuator experiment, with each other based on the maximum of the power-normalized cross correlation (ρ). For a single bolus, 3 × 3 clusters with ρ>0.8 for a subject indicate high similarity between all three trials. These can be found individually for saliva and water (both “b”), yogurt (“b”, “c”) as well as between water and yogurt (also “b”). Clusters with at least 5 entries within a 3 × 3 matrix (ρ>0.8) exist for saliva (“c”), water (“a”, “c”, and “a” vs. “b”), and yogurt (“a” vs. “b”).

Figure 9b displays the same data grouped per subject to assess subject-specific similarity beyond the swallowing task. It again highlights the correlation between water and yogurt for Subject “b” as well as some similarities between Subjects “a” and “b”. It also shows a consistently higher correlation for “b” in comparison to “a” and “c”.

**Figure 10:**
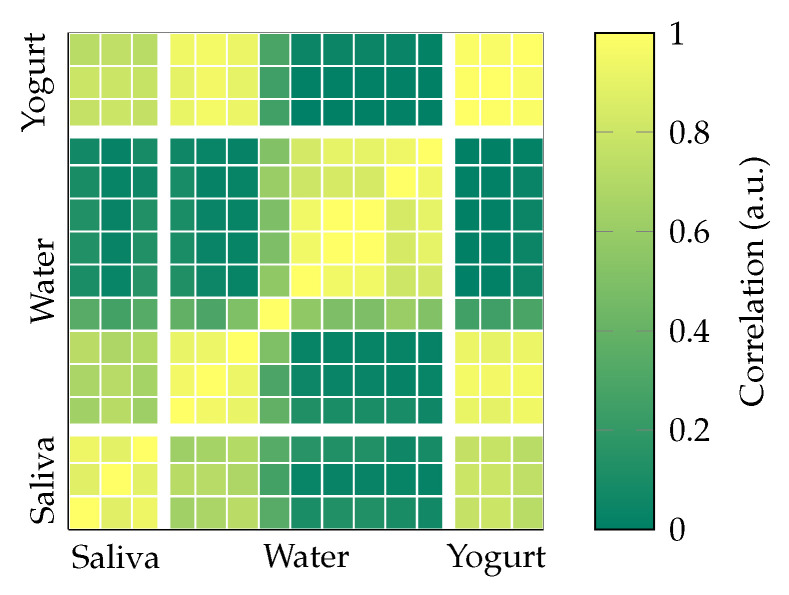
Normalized cross-correlation for both experiments with Subject “b” only.

Figure 10 results from the same methodology regarding the correlation matrix exclusively for all available signals of Subject “b”. In addition to the nine trials from the single actuator experiment, it also incorporates six trials (water only) from the dual actuator experiment. Apart from the previously described clusters for Subject “b” (saliva, water, yogurt, water vs. yogurt), there is a very large cluster for all except for the first trial of the dual actuator experiment in water. However, a similarity between the water trials for both experiments (single and dual) is not observed.

## 4. Discussion

In this paper, we evaluated the swallowing detection capability of a MI sensor with single and dual actuators. Results showed the suitability of the proposed sensor. However, the results shown in Figure 4 depend on the ability of the person to keep the position during signal acquisition because of the high susceptibility to movements. The average standard deviation of the pulse width for all subjects for almost all swallowing processes was about 11%. The calculation of the defined pulse width feature was not possible for all measurements: for instance, head movements at the end of the swallowing process produce differently shaped and highly asymmetrical deglutition-related lobes. Thus, it was not possible to calculate the pulse width for 3 of the 27 measurements in total. For further discussion, the mean over three swallowing attempts and the overall mean were used, as displayed in Figure 5 for Subject “b”. Even though the quality of matching of the reference line was different for all subjects, a distinct difference between swallowing of different consistencies was visible at least for Subjects “b” and “c”. In general, the amplitudes clearly differed between swallowing tasks. However, no pattern for the differentiation of the swallowing tasks by amplitude could be found. From the prototype signals depicted in Figure 6 it can be seen, that Subjects “a” and “c” had similar peak-to-peak amplitudes of nearly 0.5 nT, but clearly differed in duration with 27 and 19 ms. The signal of Subject “b” had a much higher amplitude of nearly 1.1 nT and in addition the highest duration of 34 ms. While the signals of Subjects “a” and “b” were nearly symmetric and Gaussian-like, the swallowing signal of Subject “c” exhibited a non-symmetric curve with a second small peak right before the main lobe. These individual-specific properties seem to appear with every swallowing trial.

Bearing in mind that the trials included in this pilot study avoid the generalization of results, we performed a general analysis of the behavior of the features per swallowing task to overcome this limitation (Table 3). The feature-related values shown in Table 3 suggest a differential pattern of the magnetic signals measured in the three swallowing tasks for the single actuator setup. The frequency-domain features (MNF, MDF, PKF, $f_{\mathrm{low}}$, $f_{\mathrm{high}}$, BW) were higher for water than for saliva and yogurt, which exhibited similar behavior; however, the RMS was lower for water, similar to MNP, Ea, and Ed4.

The RMS extracted from the MI sensor shows higher values for saliva and yogurt than for water. This is consistent with the electromyography-related literature since saliva shows a shorter duration but higher amplitude than water and is comparable to thick liquids [53]. Furthermore, the fact that amplitude is higher for thick liquid than for thin liquids is also consistent with the swallowing biomechanics [60], and it has also been reported in patients with mild dysphagia [61]. In healthy individuals, thick liquids demand more effort than thin ones, and effortful swallows have more amplitude variance than non-effortful swallows [13]. In this way, we evidenced that VAR was higher for saliva and yogurt than for water.

Like RMS and VAR, saliva and yogurt produced similar values in MNF, MDF, PKF, $f_{\mathrm{low}}$, MNP, and Ea. This is noticeable because the volume of water and yogurt was the same, i.e., 20 mL. Thus, preliminary results suggest that the proposed setup could be suitable to characterize the swallowing for different consistencies, in this case, thin vs. thick liquids. MNF, MDF, PKF, $f_{\mathrm{low}}$, $f_{\mathrm{high}}$, and BW were higher for water, which agrees with the literature: Youmans and Stierwalt [62] found that swallowing of thin liquids produced higher spectral components. However, we cannot compare the obtained values because the observed ranges are very different from the characteristic bandwidth obtained with other signals. For instance, we found that BW <1 Hz, but in swallowing sounds, the frequencies are between 400 and 1000 Hz, the bandwidth with accelerometers is below 300 Hz, or the main spectral components in surface electromyography ranges mainly between 90 and 250 Hz [13,63,64,65]. Nevertheless, these findings must be handled with care and cannot lead to definite conclusions because the measurement bandwidth of our setup is limited to 10 Hz.

In our initial hypothesis, we assumed a characteristic pattern per swallowing task, which would result in a high correlation (Figure 9a). While the results for Subject “b” per swallowing task generally show a high correlation, findings for Subjects “a” and “c” were not conclusive. The resemblance between the single and dual actuator experiments of Subject “b” (water, Figure 10) was limited. Comparison of the patterns themselves (time signal) shows an overall similarity in signal shape within tasks with variations in amplitude (Figure 4). As the main cause for this behavior, we assume differences in the relative position between the actuator and sensor are caused by changes in seating position, head posture, and actuator placement. While the applied reference line method assures a common DC offset (starting point) for the magnetic signal, it can only partly assure a repeatable start position and orientation in 3D space.

From the signals of the second actuator, the swallowing process was not as visible as for the signal of the first one, and we were not able to determine a characteristic pattern. A possible cause could be the placement of the second actuator which could be chosen differently. However, this measurement setup was mostly conducted to prove the general concept of separating both actuator signals in a frequency-division multiple access (FDMA) scheme.

Table 4 shows that features in time (RMS, VAR) are quite similar for both actuators, but frequency domain features actually differ. Despite the high standard deviation in both actuators and the low-frequency values, results suggest that MNF, MDF, PKF, and $f_{\mathrm{low}}$ were higher in Actuator A than in B. This observation disagrees with the literature which establishes that suprahyoid frequency components are higher than infrahyoid ones [66,67]. However, it is important to note that despite the actuator’s placement overlapping spatially with the infra- and suprahyoid muscles, it is not possible to establish a direct correlation between electrophysiological activity measured by electromyography and the variations of the magnetic field produced by swallowing-related movements. To the best of our knowledge, this is the first study in which an MI sensor is used to detect the swallowing function, so the reported results cannot be compared with the literature directly. The most related study is the one performed by Monaco et al. [68] in which the authors used the change of the magnetic flux of a kinesiograph to detect the mandibular movement during swallowing. This movement is related to the activation of the suprahyoid muscle group, in the same place as our Actuator B in the dual-sensor experiment. However, the kinesiographic measurements were intended to describe mandibular movements, which are not of interest in the current work. Thus, the closest relation could be made between Actuator A in single and dual actuator setups and the patterns observed in the accelerometry-based cervical auscultation. Even though the accelerometer was placed at the same point to measure the movement of the cricoid cartilage [69], accelerometry discriminates between axes, especially anterior–posterior and superior–inferior movements, but our setup captures magnetic variations produced by changes of the relative position between the actuator and the MI sensor. So, both approaches provide different information, and the results in terms of characterization are not comparable. This opens an opportunity to research the characterization of the magnetic signals from MI sensors in healthy populations and its comparison with patients with swallowing disorders.

**Figure 11:**
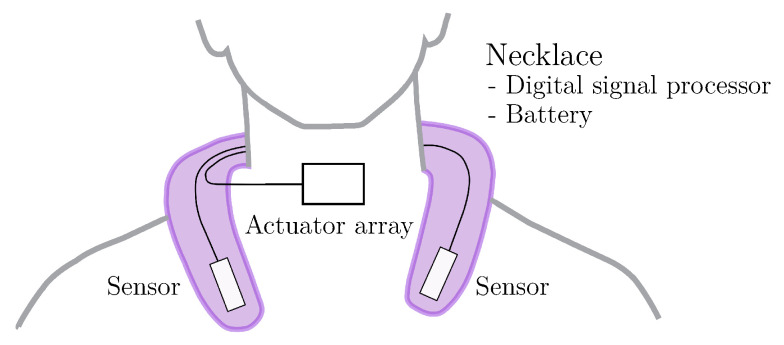
Concept of a wearable necklace setup.

Generally, the applied setup might be improved in several ways. For instance, the attachment of the body-worn actuators and corresponding wiring with adhesive tape limited the ability for a robust measurement routine due to weight and soft-tissue artifacts. On the other hand, a fully wearable solution as desired for home assessment would also require body-worn sensors and a battery-powered platform for signal generation and processing. We are currently working on a necklace-based setup (Figure 11) to achieve this goal. The application of energy-efficient cantilever actuators instead of coils might be also be beneficial in such a scenario.

The measurements of Actuator A show sufficient accordance between single and dual actuator experiments (cf. Figure 4 and Figure 7) based on visual inspection. Therefore, we conclude that the simultaneous operation of multiple actuators by FDMA is feasible, which paves the way for more complex operation schemes. Multiple actuators might be employed in several anatomical regions for a comprehensive assessment (in combination with physiological/clinical variables). Detailed mapping of the whole throat area (array approach) might also be beneficial to capture spatial variations with high resolution. Lack of reproducibility might be overcome by defining a more accurate reference position or rejecting movement artifacts (based on multiple actuators or sensors). Furthermore, the use of triaxial magnetic sensors and actuators might be considered, which could enable the reconstruction of unambiguous position and orientation signals instead of magnetic signals.

Finally, validation with the reference methods, e.g., videofluoroscopy, must be performed in order to establish underlying relationships between changes in the signal detected by the MI sensor and physiological movements observed during the swallowing. The advantage of our setup is that the actuators are placed in positions that allow the lateral visualization of the swallowing during the videofluoroscopy. Further studies must address questions such as: are there MI signal-related differences between healthy and dysphagic patients? How significant are the differences between consistencies and volumes? Is it possible to differentiate swallows by gender? Such questions may also be more easily addressed through increased database sizes.

## 5. Conclusions

In this work, we proposed the use of a novel magnetic setup to assess the swallowing process based on a magnetoimpedance sensor and body-worn cantilevers actuators. This proof-of-concept shows that the proposed setup is capable of detecting swallows from different consistencies, i.e., thin liquid, thick liquid, and saliva. We performed a visual inspection, signal characterization (time, frequency, and time–frequency), and correlation analysis to highlight (and prospectively discriminate) swallows per bolus type. There was a characteristic pattern of the amplitude according to the consistency; yogurt achieved greater amplitude than water, which retrieved greater amplitude than saliva. We found that amplitude-based measures increased clearly during the bolus intake, and there was also an energy increase observed in the time–frequency domain during swallowing regardless of the consistency. In the dual actuator experiment, we successfully applied a secondary actuator to demonstrate the potential for setup improvements such as an increased spatial resolution. We obtained similar time domain features for both actuators, but different frequency domain ones, and a lack of characteristic patterns in correlation plots, suggesting that multiple actuators provide complementary information. Although further validations should be performed, the preliminary results indicate suitability as a non-invasive and quantitative method for dysphagia screening and bio-feedback for diagnosis and therapy.

## Figures and Tables

**Table 1:** Demographic data of participants.

Subject	Sex	Age
a	Male	36
b	Female	24
c	Male	50

**Table 2:** Mathematical formulations of features.

Feature	Equation	Feature	Equation
VAR	$\frac{1}{N-1}\sum_{i=1}^{N}x_{i}^{2}$	RMS	$\sqrt{\frac{1}{N}\sum_{i=1}^{N}x_{i}^{2}}$
MNP	$\frac{1}{M}\sum_{j=1}^{M}P\left(f_{j}\right)$	MNF	$\left.\sum_{j=1}^{M}f_{j}P\left(f_{j}\right)/\sum_{j=1}^{M}P\left(f_{j}\right)\right.$
MDF	$\sum_{j=1}^{MDF}P\left(f_{j}\right)=\frac{1}{2}\sum_{j=1}^{M}P\left(f_{j}\right)$	PKF	$\underset{f}{\mathrm{argmax}}\left\{P\left(f\right)\right\}$
Ea	$\sum_{i}{\left\{cA\left(i\right)\right\}}^{2}$	Edj	$\sum_{i}{\left\{cDj\left(i\right)\right\}}^{2}$

*x_i_*: *i*-th sample of the signal; *N*: length of the signal; *M*: length of the power spectral density (PSD); *P*(*f_j_*): PSD evaluated at the *j*-th frequency *f_j_*; *cA*(*i*) and *cDj*(*i*): *i*-th sample of the approximation and *j*-th detail coefficients, respectively.

**Table 3:** Features grouped per swallowing task for the single actuator experiment. Values provided in mean ± standard deviation. Domains are separated by grey lines.

Feature	Saliva	Water	Yogurt
Amplitude (nT)	0.67 ± 0.08	0.66 ± 0.13	0.85 ± 0.13
Pulse width (s)	0.28 ± 0.01	0.26 ± 0.03	0.29 ± 0.04
RMS (nT)	0.28 ± 0.18	0.19 ± 0.07	0.30 ± 0.08
VAR (nT^2^)	0.10 ± 0.12	0.04 ± 0.03	0.09 ± 0.05
MNF (Hz)	0.83 ± 0.20	1.03 ± 0.18	0.73 ± 0.13
MDF (Hz)	0.56 ± 0.21	0.85 ± 0.25	0.47 ± 0.16
PKF (Hz)	0.43 ± 0.29	0.67 ± 0.38	0.33 ± 0.10
$f_{\mathrm{low}}$ (Hz)	0.24 ± 0.05	0.34 ± 0.16	0.24 ± 0.05
$f_{\mathrm{high}}$ (Hz)	0.73 ± 0.34	1.08 ± 0.51	0.51 ± 0.08
BW (Hz)	0.50 ± 0.31	0.73 ± 0.47	0.27 ± 0.08
MNP (nT^2^Hz^−1^)	0.06 ± 0.07	0.02 ± 0.02	0.06 ± 0.03
Ea (dB)	12.68 ± 5.49	9.93 ± 3.61	14.57 ± 2.23
Ed4 (dB)	−7.78 ± 5.04	−7.69 ± 4.44	−5.97 ± 3.58

**Table 4:** Features obtained in the dual actuator experiment with water for Subject “b”. Values provided in mean ± standard deviation. Domains are separated by grey lines.

Feature	Actuator A	Actuator B
RMS (nT)	0.21 ± 0.05	0.23 ± 0.13
VAR (nT^2^)	0.05 ± 0.02	0.06 ± 0.07
MNF (Hz)	1.00 ± 0.24	0.64 ± 0.23
MDF (Hz)	0.74 ± 0.30	0.38 ± 0.21
PKF (Hz)	0.42 ± 0.29	0.33 ± 0.24
$f_{\mathrm{low}}$ (Hz)	0.31 ± 0.18	0.21 ± 0.13
$f_{\mathrm{high}}$ (Hz)	0.60 ± 0.32	0.56 ± 0.19
BW (Hz)	0.30 ± 0.17	0.35 ± 0.13
MNP (nT^2^Hz^−1^)	0.03 ± 0.01	0.04 ± 0.05
Ea (dB)	11.35 ± 2.15	11.78 ± 4.76
Ed4 (dB)	−7.78 ± 1.45	−11.20 ± 3.42

## Data Availability

Result data is available online as part of the Kiel-Medellín Magnetic Swallowing Assessment Database. https://biomagnetic-sensing.de/index.php/data-bases/datasets/magnetic-swallowing-database, accessed on 27 March 2023.

## Abbreviations

The following abbreviations are used in this manuscript:

MEMS	MicroElectroMechanical Systems
ISIT	Institute for Silicon Technology
CA	Cervical Auscultation
IMU	Inertial Measurement Unit
MR	MagnetoResistive
MI	MagnetoImpedance
ME	MagnetoElectric
PCB	Printed Circuit Board
ABS	Acrylonitrile Butadiene Styrene
DC	Direct Current
BNC	Bayonet Neill–Concelman
IIR	Infinite Impulse Response
RMS	Root Mean Square
VAR	Variance
MNF	Mean Frequency
MDF	MeDian Frequency
MNP	MeaN Power
PKF	Peak Frequency
BW	BandWidth
PSD	Power Spectral Density
CRC	Collaborative Research Center
